# A Novel Microbial Culture Chamber Co-cultivation System to Study Algal-Bacteria Interactions Using *Emiliania huxleyi* and *Phaeobacter inhibens* as Model Organisms

**DOI:** 10.3389/fmicb.2018.01705

**Published:** 2018-07-30

**Authors:** Mariane S. Thøgersen, Jette Melchiorsen, Colin Ingham, Lone Gram

**Affiliations:** ^1^Department of Biotechnology and Biomedicine, Technical University of Denmark, Kongens Lyngby, Denmark; ^2^Hoekmine BV, Utrecht, Netherlands

**Keywords:** co-culture, Emiliania huxleyi, Phaeobacter inhibens, microbial culture chamber, biofilm, qPCR

## Abstract

Our understanding of microbial natural environments combines *in situ* experimentation with studies of specific interactions in laboratory-based setups. The purpose of this work was to develop, build and demonstrate the use of a microbial culture chamber enabling both *in situ* and laboratory-based studies. The design uses an enclosed chamber surrounded by two porous membranes that enables the comparison of growth of two separate microbial populations but allowing free exchange of small molecules. Initially, we tested if the presence of the macroalga *Fucus vesiculosus* inside the chamber affected colonization of the outer membranes by marine bacteria. The alga did indeed enrich the total population of colonizing bacteria by more than a factor of four. These findings lead us to investigate the effect of the presence of the coccolithophoric alga *Emiliania huxleyi* on attachment and biofilm formation of the marine bacterium *Phaeobacter inhibens* DSM17395. These organisms co-exist in the marine environment and have a well-characterized interdependence on secondary metabolites. *P. inhibens* attached in significantly higher numbers when having access to *E. huxleyi* as compared to when exposed to sterile media. The experiment was carried out using a wild type (wt) strain as well as a TDA-deficient strain of *P. inhibens*. The ability of the bacterium to produce the antibacterial compound, tropodithietic acid (TDA) influenced its attachment since the *P. inhibens* DSM17395 wt strain attached in higher numbers to a surface within the first 48 h of incubation with *E. huxleyi* as compared to a TDA-negative mutant. Whilst the attachment of the bacterium to a surface was facilitated by presence of the alga, however, we cannot conclude if this was directly affected by the algae or whether biofilm formation was dependent on the production of TDA by *P. inhibens*, which has been implied by previous studies. In the light of these results, other applications of immersed culture chambers are suggested.

## Introduction

Microbiologists have for decades been focused on isolation and growth of pure bacterial cultures. However, very few microorganisms grow axenically and a major and logical research trend is to study functionality of co-cultured organisms or even communities (D’Alvise et al., 2012; Stewart, 2012; Boon et al., 2014; Goers et al., 2014; Ramanan et al., 2016). To support these aims, techniques are being developed for microbial co-culture studies and these are also being used as means of increasing culturability of microorganisms from natural samples (Kaeberlein et al., 2002; Morris et al., 2008; Nichols et al., 2010; Park et al., 2011; Ling et al., 2015) or as systems for co-culturing of already isolated microorganisms in order to, e.g., induce otherwise silent biosynthetic gene clusters of one organism by another (Shi et al., 2017; Wakefield et al., 2017). Examples of such techniques include diffusion chambers such as the iChip (Nichols et al., 2010) where enclosed chambers porous for small molecules are inoculated with an environmental sample without any nutrients present, sealed in agarose enclosed between two polycarbonate membranes, and placed back in the environment or environmental sample to incubate. The membranes allow for nutrient, quorum sensing molecules and other small naturally occurring compound to diffuse into the chamber. Several variants of such culture chambers exist including the microbial culture chip, where bacteria grow on a membrane subdivided into hundreds of small compartments. The membrane is placed on a liquid or an agar surface and micro-colonies develop in each compartment (Ingham et al., 2007; Ingham and van Hylckama Vlieg, 2008; Catón et al., 2017).

Microbial culture chambers are generally single chambers connected to the environment by porous membranes (Kaeberlein et al., 2002; L’Haridon et al., 2016). These are built to enrich a complex mixture of microorganisms. Current designs are not orientated toward the formation of biofilms and do not allow for evaluation of why a specific population of microorganisms are cultured. The concept of an asymmetric culture chamber was briefly described by L’Haridon et al. (2016). This device was designed to allow for experiments to be conducted within the natural environment with the focus on biofilm formation. In the present study, we have manufactured significant numbers of such chambers and applied them to study interactions in controlled bacterial/algal co-culture systems.

Communities of marine bacteria are known to live in association with and colonize the surface of macro algae. Some algae such as *Fucus vesiculosus*, produce a range of chemical compound to attract a specific mixed bacterial community (Wahl, 1989; Lachnit et al., 2010, 2013; Saha et al., 2011). Others live in more species specific interdependent relationships. We have, over the past decade, studied the interactions between the marine bacterium, *Phaeobacter inhibens* and other bacteria, especially those that are fish pathogenic (Prol et al., 2009; D’Alvise et al., 2012; Prol García et al., 2014; Grotkjær et al., 2016; Porsby and Gram, 2016). *P. inhibens* is a common part of the microbiota in marine aquaculture and has potential as a fish probiotic due to production of a small antibacterial compound, tropodithietic acid (TDA) (Grotkjær et al., 2016; Porsby and Gram, 2016). In the marine environment, *P. inhibens* is found in biofilms (Gram et al., 2015) or in association with the coccolitophoric microalgae *Emiliania huxleyi* (Seyedsayamdost et al., 2014; Segev et al., 2015; Wang et al., 2016). The bacterium is able to switch from mutualist to parasite in response to the growth and life cycle of *E. huxleyi* (Seyedsayamdost et al., 2011a,b, 2014, Sonnenschein et al., 2018). The relationship is mutualistic when the algae produce dimethylsulfoniopropionate (DMSP), which provides a source of sulfur and carbon for the bacteria, and the bacteria produce growth hormones and anti-bacterial compounds for the algae in form of phenylacetic acid and TDA, respectively. The relationship becomes parasitic, when the algae reach stationary phase and secrete *p*-coumaric acid (pCA) as well as sinapic acid, which are potential senescence signals, to which the bacteria react by activating otherwise silent biosynthetic pathways that encode the algaecidal roseobacticides and roseochelins, respectively (Seyedsayamdost et al., 2014; Wang and Seyedsayamdost, 2017).

*Phaeobacter inhibens* is an excellent biofilm former (Bernbom et al., 2011; Gram et al., 2015) and is able to colonize both biotic and abiotic surfaces while outcompeting other bacteria (Lutz et al., 2016; Zhao et al., 2016). We therefore speculate that the presence of *E. huxleyi*, due to their common co-existence *in vivo* and their known exchange of bioactive molecules, could stimulate attachment and biofilm formation of *P. inhibens.* To enable studies of such co-culture interactions, we developed and applied the microbial culture chamber and propose that it can also be used in future studies for enhancement and development of new antibacterial compounds.

## Materials and Methods

### Overview of Co-cultivation Chamber

**FIGURE 1:**
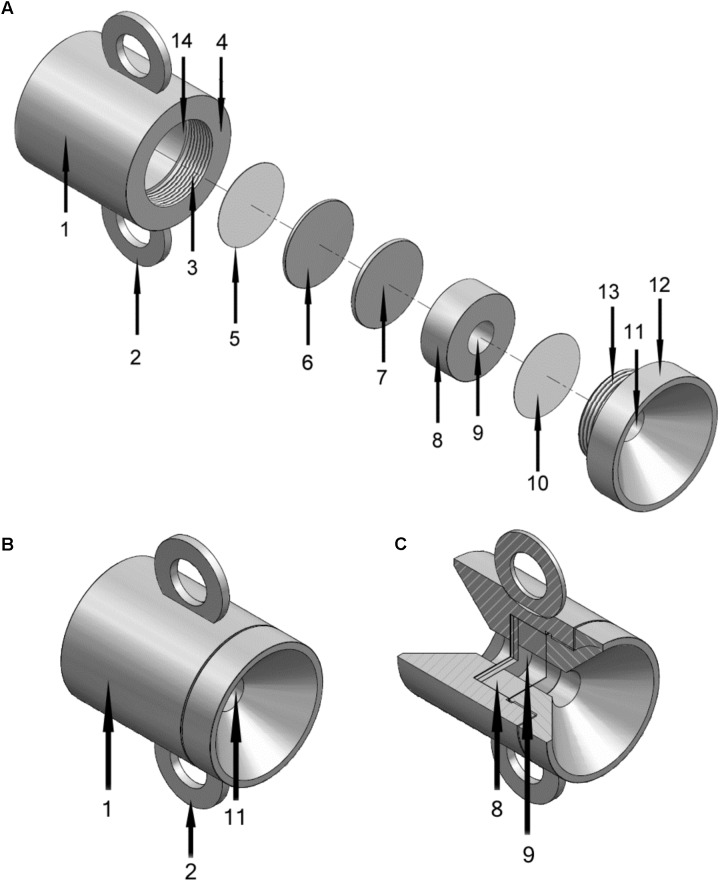
Schematics of the microbial culture chamber made from stainless steel, total height 5 cm. **(A)** Disassembled view. 1, Main housing of culture chamber; 2, Handle (one of two) for attachment to structure, ship, chain or other anchor point; 3, Screw thread for closure (with 13); 4, Outer lip of main housing that fits to screw cap (12); 5, Porous membrane (control membrane) exposed to environment but prevented from exposure to central chamber (9) by 6 and 7; 6, Solid metal spacer A; 7, Solid metal spacer B (solid spacer B can be removed to accommodate thicker membranes); 8, Ringed structure that defines central chamber (with 7 and 10); 9, Central chamber; 10, Porous membrane exposed to chamber one side and exterior (via 11) the other (control membrane); 11, Hole in screw cap (12) exposing 10 to environment; 13, Screw thread for closure (with 3); 14, Interior of 1 where items 5 to 10 fit into. **(B)** View of assembled chamber with component numbers as described for panel A. **(C)** Cut away view of assembled chamber.

**FIGURE 2:**
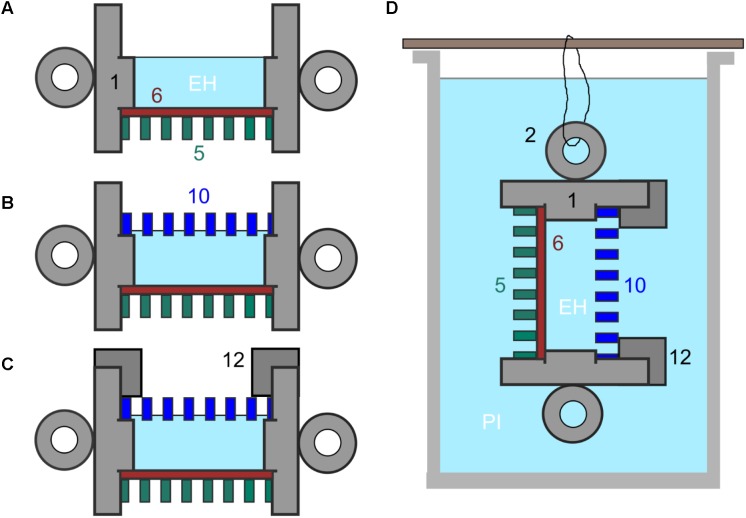
**(A–C)** Set up of culture chamber from autoclaved components plus sterile membranes with numbering as indicated in **Figure 1**. **(A)** Add control membrane (11) using sterile tweezers and then at least one spacer (6) ensuring tight fit then fill central chamber with culture medium and, in the experiments described here, with *E. huxleyi* (EH). **(B)** Add experimental membrane (10), avoiding air bubbles. **(C)** Screw down cap (12). **(D)** Final culture chamber deployment, shown suspended in a beaker of f/2 medium inoculated with *P. inhibens* (PI) from one of the handles (2).

For co-cultivation, we used the microbial culture chamber, a stainless-steel device for *in situ* culture and enrichment of microorganisms (**Figure 1**). The culture chamber has a central chamber connected to the outside through a porous membrane (supplied by the user so material and pore size is optional) allowing microorganisms to be cultured on the external surface or the membrane with access to diffusible molecules from the inner chamber (experimental membrane). A second membrane is sealed from access to the chamber by a solid plate and acts as a control (control membrane) (**Figure 2**). A second solid plate acts as a spacer and can be removed to accommodate thicker stacks of membranes, if required (**Figure 1**). A thicker stack of membranes on the outer side of the chamber can be deployed and has the function of fine tuning the rate of diffusion between environment and inner chamber.

This set up allows for co-culturing of two organisms while keeping them physically separated by the membrane. Alternatively, this device makes it possible to fill the inner chamber with, e.g., an antimicrobial compound and investigate how an outside microbial culture or mixed community responds to that compound. The idea is that microorganisms from a given environment or pure culture will be able to attach and grow and form a biofilm on the outer surface of the membranes while being exposed to the outside environment as well as to compounds diffusing out from the inner chamber. The solid plates can be supplemented with an exterior porous membrane to serve as a control, where the membrane is only exposed to the outside environment without being in contact with the inner chamber.

### Sterilization and Preparation of Chamber

The individual components of the chamber (**Figure 1**) can be sterilized by autoclaving although immersion in 70% (w/v) ethanol is sufficient for most purposes. The chamber components were autoclaved prior to assembly in a laminar flow hood (**Figure 2A**) and then the set is up completed, as described in **Figure 2** and in individual experiments, below.

### Application of the Device in Co-culture

The chamber was assembled with one polycarbonate membrane (25 mm Ø, 0.2 μm, GE Water & Process Technologies, PA, United States, Cat.No. K02CP02500) sealed from the inner chamber with two metal plates (**Figure 1**). The inner chamber was filled with 1 ml 7-days culture of *E. huxleyi* CCMP2090 (2 × 10^6^ cells/ml) or 1 ml f/2 medium with 3% IO (negative control) and topped with two 0.2 μm polycarbonate membranes (as above) and closed with the top part of the culture chamber.

The assembled chamber was submerged in 500 ml f/2 medium with 3% IO, which was inoculated with 10^6^ cells/ml *P. inhibens* wt or *P. inhibens* Δ*tdaB* with gentle stirring (200 rpm on a magnet stirrer) at RT. The experiment was carried out in two independent experiments, each in triplicate, and incubations were for 24, 48, and 96 h. Tracking beads (0.5 μm fluorescein labeled polycarbonate microbeads, Polysciences, DE) were inserted into the central chamber (10^6^/ml) in control experiments for leakage. After incubation, the chambers were dissembled. Three experimental membranes and three control membranes were randomly selected for SYBR Gold staining to evaluate bacterial biofilm formation by epifluorescence microscopy. In the same manner, three experimental membranes and three control membranes were selected for DNA extraction for subsequent qPCR to quantify the number of bacteria attached (below).

### Application of the Device for Microbial Enrichment

The chamber was assembled as described above except that (A) Porous aluminum oxide membranes (25 mm diameter, pore size on outer surface 20 nm, porosity 30%, from SPI Supplies, United States) were used in place of polycarbonate membranes, and (B) a 0.6 g (wet weight) piece of macroalgae (Bladder Wrack, *F. vesiculosus*, from Rotterdam Harbour, NL) was placed the inner chamber. The algal fragment was washed in 5 ml of sterile artificial sea water. The assembled chamber was suspended in a low nutrient environment, i.e., sterile artificial sea salt medium (3.5% w/v Sigma, NL). The artificial sea water was spiked with 1 ml of medium used to wash the algal fragment. After a week, microorganisms from the outer surfaces of the membranes were then quantified after staining with SYTO9 and hexidium iodide and imaging as described below.

### Testing Chamber Permeability to Diffusible Molecules

The chamber was assembled as described above except that it was loaded with LB containing 5 × 10^6^ cfu/ml *E. coli* CTX3 (*E. coli* DH5alpha with pACTEM1 plasmid, IPTG-inducible TEM-1 β-lactamase; MIC for cefotaxime of 0.25 μg/ml) or CTX6 (similar to strain CTX1 but MIC > 512 μg/ml) (Finkelshtein et al., 2015). The exterior of the chamber was surrounded with L-broth either in the absence of any antibiotic or with cefotaxime (10 μg/ml) or rifampicin (20 μg/ml) and incubated at 37°C for 36 h. For all experiments involving cefotaxime, 50 μm IPTG was present in all media to induce the plasmid encoded resistance to this antibiotic. At the end of each experiment viable counts on L-agar were made for the bacteria in the central chamber to determine the degree to which the external antibiotics were affecting the growth of *E. coli* in the internal chamber. Additional experiments were performed in the absence of microorganisms using phosphate buffered saline; by loading the inner chamber with 1 mm fluorescein (Sigma, NL). At different time points 10 μl aliquots from the buffer surrounding the culture chambers were spotted on microscope slides, dried and observed by fluorescence microscopy to estimate the concentration against known standards.

### Application of the Device for Co-culture

*Emiliania huxleyi* (CCMP2090) (Hay et al., 1967) was cultured in f/2 medium [0.882 mM NaNO_3_, 0.0362 mM NaH_2_PO_4_⋅H_2_O, 0.106 mM Na_2_SiO_3_⋅9H_2_O, 1 ml/l trace metal solution, and 0.5 ml/l vitamin solution] (Guillard and Ryther, 1962; Guillard, 1975) with 3% Instant Ocean (IO) (Aquarium Systems, Sarrebourg, France) at 16°C with daylight for 7 days until a cell count of approx. 2 × 10^6^ cells/ml was reached. *P. inhibens* DSM17395 (hereafter, *P. inhibens* wt) (Martens et al., 2006) and *P. inhibens* DSM17395 Δ*tdaB* (hereafter, *P. inhibens* Δ*tdaB*) (kindly provided by Dr Muhammad Seyedsayamdost, Harvard Medical School, Boston, MA, United States) was cultured in 1/2YTSS [2 g/l Bacto Yeast extract, 1.25 g/l Bacto Tryptone, and 20 g/l Sigma Sea Salts] (Sobecky et al., 1997; D’Alvise et al., 2012) at RT for 24 h with agitation (200 rpm). After incubation, *P. inhibens* cells were harvested (6 min at 6000 rpm), washed twice in f/2 medium with 3% IO, and resuspended to approx. 10^8^ cells/ml in f/2 medium with 3% IO and 5 mM NH_4_Cl.

### Staining of Membranes With Fluorogenic Dyes

The outermost membrane from the open end of the chamber (experimental membrane) and the membrane from the blinded end (control membrane) were removed from the culture chamber and placed on top of 30 μl SYBR Gold staining solution in a Petri dish followed by 15 min incubation in the dark. The membranes were rinsed by placing on top of 100 μl MilliQ H_2_O, and then dried on a piece of paper towel for 30 min. in the dark. The membranes were mounted on Superfrost glass slides (Thermo Scientific, MA, United States) with 10 μl mounting buffer [100 μl PBS, 100 μl 87% glycerol, 2 μl 10% p-phenylene-diamine] below and on top. A cover slip was mounted on top. Visualization was carried out using epifluorescence microscopy with an Olympus BX51 fluorescence microscope with a 460–490 nm excitation membrane and a > 510 nm barrier membrane. A similar procedure was used for SYTO9 and hexidium iodide staining microorganisms on porous aluminum oxide membranes as previously described (Ingham et al., 2007) followed by quantification in ImageJ version 1.45S (Schneider et al., 2012).

### DNA Extraction From Membranes

DNA was extracted from the membranes using phenol:chloroform:isoamyl alcohol (modified from Boström et al., 2004). In brief, the membranes were submerged in 1 ml lysis buffer [1 mg/ml lysozyme, 40 mM EDTA, 50 mM Tris pH 8.2, and 0.75 M sucrose]. The membranes were incubated in the lysis buffer for 30 min at 37°C with agitation. Sodium dodecyl sulfate (SDS) was added to a final concentration of 1% together with Proteinase K (Sigma, P6556) to a final concentration of 0.1 mg/ml and samples were incubated at 55°C overnight with constant agitation. Membranes were removed and washed with 500 μl TE 10:1 buffer. The lysate and the TE buffer were pooled, transferred to a clean tube, and DNA extraction was carried out using an equal volume of phenol:chloroform:isoamyl alcohol (25:24:1, vol:vol:vol) using standard procedures, and finally the DNA pellet was resuspended in 50 μl MilliQ H_2_O.

### qPCR Procedures

qPCR amplifications were performed on 1–10 μl template DNA (10 ng) using SYBR Green PCR Master Mix (Applied Biosystems, CA, United States, Cat.No. 4309155) with ROX (6-carboxy-X-rhodamine) as a reference dye, in a 25 μl reaction volume containing 50 nM of each primer. The primers used were designed within the 16S gene of *P. inhibens* (forward primer: 5′- TGCCGCGTGAGTGATGAA-3′, reverse primer: 5′- ATTCCGAACAACGCTAACC-3′) to give a fragment of 140 bp. The PCR amplification (10 min at 95°C followed by 40 cycles of three steps consisting of 15 s at 95°C, 1 min at 58°C, and 1 min at 72°C) was performed with an Mx3005P qPCR thermal cycler (Stratagene, La Jolla, CA, United States). All samples were subjected to melting curve analysis.

Four individual cultures of *P. inhibens* wt were grown in 150 ml 1/2YTSS for 4 days at RT and 200 rpm. The cultures were 10-fold serially diluted, and DNA was extracted from duplicate samples of each dilution using the Nucleospin Tissue Kit (Machery-Nagel, Düren, Germany, Cat.No. M740952), and *C_T_* (cycle threshold) was determined in real-time PCR procedure as described above. CFU/ml was determined on 1/2YTSS agar for each dilution and a standard curve relating cell number to qPCR *C_T_* values developed by linear regression.

### Statistical Analyses

*C*_t_ values were transformed to log (CFU/ml) and CFU/ml based on the qPCR standard curve. Normality tests were performed in Minitab (Minitab 18, Minitab Inc.). Significance of number of attached cells between log(CFU/ml) of *P. inhibens* WT and *P. inhibens* Δ*tdaB* with either *E. huxleyi* or f/2 medium with open or blinded chamber over time (24, 48, and 96 h) were determined using one-way ANOVA *post hoc* Tukey test in Minitab as the data were normally distributed (*P* > 0.05). Individual samples were compared using paired *t*-test (confidence level: 95.0) in Minitab. Data that were not normally distributed were transformed to fit a normal distribution using Johnson transformation (*P*-value to select best fit = 0.10) within Minitab before ANOVA analysis was carried out.

## Results

### Biofilm Formation Enriched by the Presence of *Fucus vesiculosus*

**FIGURE 3:**
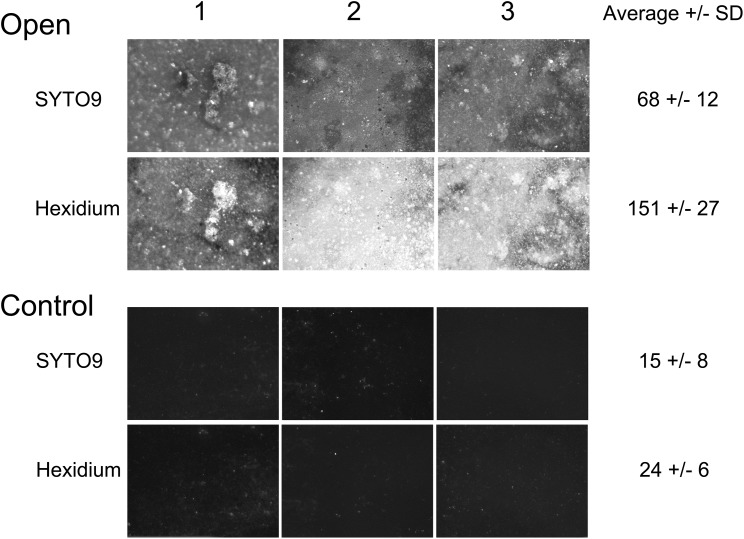
Randomly selected areas (2.5 mm × 3 mm) of PAO membranes from three culture chambers (both experimental and control membranes) stained with both SYTO9 and hexidium iodide and imaged by fluorescence microscopy. The average fluorescence intensity +/− SD after deduction of background was calculated from each set of three replicates.

After incubation for a week, the outer surface of the PAO membranes (both experimental and control membranes) were colonized with microorganisms that could be visualized by staining with SYTO9 and hexidium iodide (**Figure 3**). Enrichment of the biofilm was observed on the open, experimental membrane relative to the outer surface of the PAO membrane without a connection to the central chamber (the closed membrane). The enrichment factor was an average 4.4-fold for the SYTO staining sub-population and 6.4-fold for hexidium iodide. This suggests that nutrients originating from *F. vesiculosus*, either directly leaking from damaged cells or as a result of microbial decomposition, was able to influence a nearby community of microorganisms on the other side of a porous membrane. This result suggested it would be possible to look at a more specific microbial interaction using the culture chamber, as described below.

### The Culture Chamber Is Permeable to Antibiotics and Fluorescein

Incubation of *E. coli* CTX3 or CTX6 in the central chamber lead to growth to 1 to 3 × 10^8^ cfu/ml, suggesting good permeability to oxygen. When 10 μg/ml Ctx was present in the external growth medium then the growth of the more sensitive strain (CTX3) was inhibited (recovered CFU < 10^6^/ml from the central chamber after 36 h) whilst the resistant strain (CTX6) was not inhibited by the antibiotic (recovered CFU > 10^8^ cfu/ml). However, both strains were inhibited by rifampicin (recovered CFU < 10^6^ cfu/ml). These data suggest that the two chemically unrelated antibiotics could enter the growth chamber at effective doses. Additionally, the dye fluorescein, loaded into the chamber, was found to approach equilibration within 8 h with detectable fluorescein found after 1 h. It was concluded that “small” molecules (molecular mass 300–800 g/mol) could move across the experimental membrane.

### Attachment of *P. inhibens* in Co-culture With *E. huxleyi* Evaluated by Epifluorescence Microscopy

**FIGURE 4:**
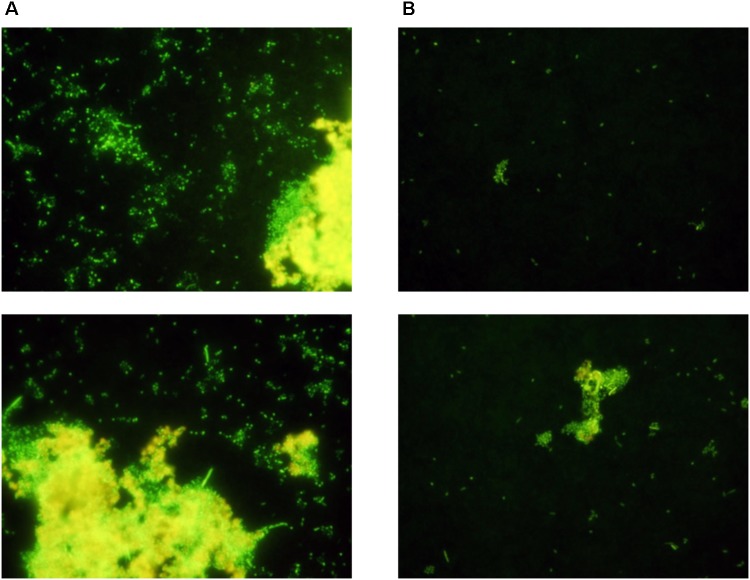
Randomly selected areas of SYBR stained membranes from co-cultivation of *E. huxleyi* with *P. inhibens* DSM17395. **(A)** Experimental membranes. **(B)** Control membranes.

**FIGURE 5:**
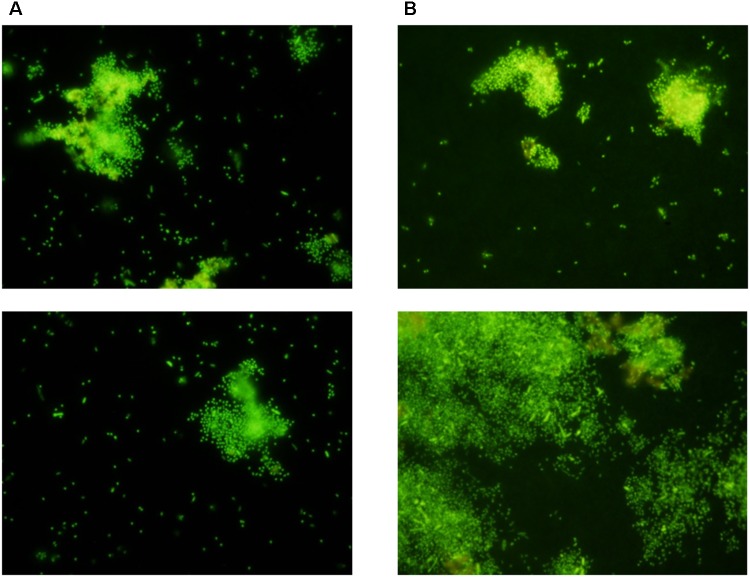
Random sections of SYBR stained membranes from negative controls with sterile f/2 medium in the inner chamber and *P. inhibens* DSM17395 outside the chamber. **(A)** Experimental membranes. **(B)** Control membranes.

The number of attached *P. inhibens* on the experimental and control membranes was evaluated by SYBR Gold staining and epifluorescence microscopy after 48 h of co-cultivation with *E. huxleyi* inside the culture chamber and *P. inhibens* wt on the outside in f/2 medium. There were more attached single cells and formation of large plaques on the outside of the experimental membranes (**Figure 4**), where *P. inhibens* had access to *E. huxleyi* compounds diffusing from the inner chamber, than on the control membranes where *P. inhibens* would not have direct access to *E. huxleyi* compounds from the inner chamber. The visual difference as detected by epifluorescence microscopy between the experimental and the control membranes were less pronounced in the negative controls, where the inner chamber contained sterile f/2 medium. A mixture of single cells and biofilm plaques were observed on both experimental and control membranes (**Figure 5**).

### Attachment of *P. inhibens* in Co-culture With *E. huxleyi* Evaluated by qPCR

**FIGURE 6:**
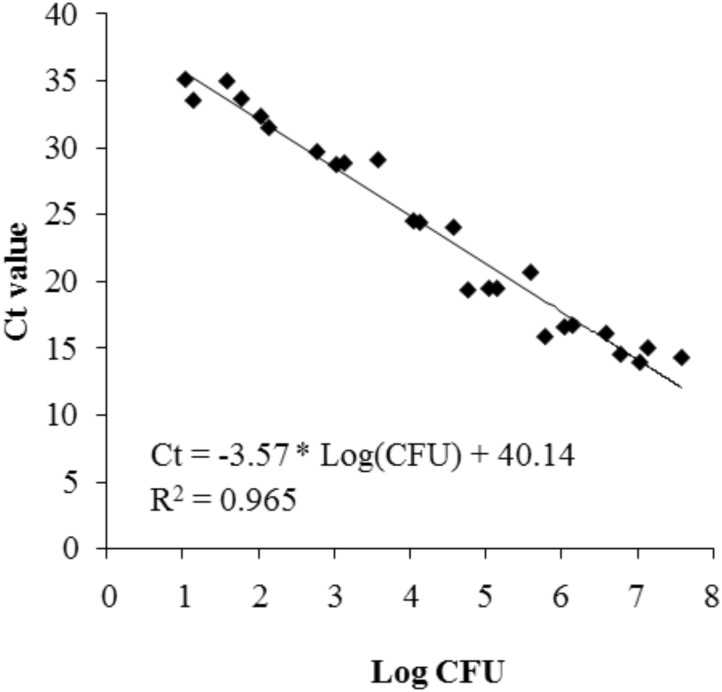
Standard curve representing the correlation between log(CFU/ml) of *P. inhibens* DSM17395 and the *C*_t_ value detected in the real-time qPCR procedure.

The standard curve relating log(CFU/ml) of *P. inhibens* to *C*_t_ values of the qPCR protocol was linear with a correlation coefficient (R^2^) of 0.965 (**Figure 6**). The attachment of *P. inhibens* wt or *P. inhibens* Δ*tdaB* to control membranes or to experimental membranes exposed to either *E. huxleyi* or f/2 medium was analyzed by qPCR measurements, and the standard curve was used to transform *C*_t_ values to log (CFU/ml).

**FIGURE 7:**
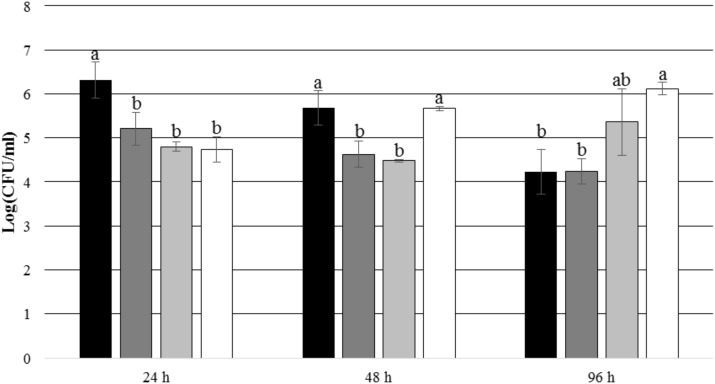
Number of attached *P. inhibens* wt and *P. inhibens* Δ*tdaB* with *E. huxleyi* is present inside the chamber. Grouping based on ANOVA *post hoc* Tukey test. Groups within each sampling time that do not share a letter are significantly different (24 h: *P* = 0.001; 48 h: *P* = 0.000; 96 h: *P* = 0.003). 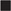 *P. inhibens* wt with *E. huxleyi*, experimental membranes; 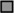 *P. inhibens* wt with *E. huxleyi*, control membranes; 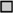 *P. inhibens*Δ*tdaB* with *E. huxleyi*, experimental membranes; 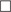 *P. inhibens*Δ*tdaB* with *E. huxleyi*, control membranes.

**FIGURE 8:**
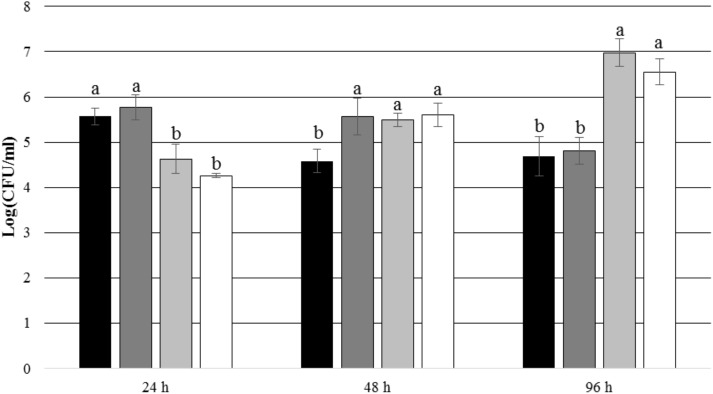
Number of attached *P. inhibens* wt and *P. inhibens* Δ*tdaB* with f/2 medium inside the chamber. Grouping based on ANOVA *post hoc* Tukey test. Groups within each sampling time that do not share a letter are significantly different (24 h: *P* = 0.000; 48 h: *P* = 0.006; 96 h: *P* = 0.000). 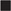 *P. inhibens* wt with f/2 medium, experimental membranes; 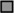 *P. inhibens* wt with f/2 medium, control membranes; 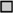 *P. inhibens ΔtdaB* with f/2 medium, experimental membranes; 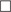 *P. inhibens ΔtdaB* with f/2 medium, control membranes.

After 24 and 48 h of incubation, the TDA-producing *P. inhibens* wt attached to the experimental membranes in significantly higher numbers than the TDA-negative mutant *P. inhibens* Δ*tdaB* when *E. huxleyi* was present inside the chamber (*p* = 0.028 for 24 h and *p* = 0.039 for 48 h). In contrast, the numbers of attached *P. inhibens* Δ*tdaB* were higher than the numbers of attached *P. inhibens* wt after 96 h (*p* = 0.014) (**Figures 7**, **8**). This indicates that production of TDA affects initial attachment and biofilm formation, and that it over time becomes inhibitory for biofilm formation.

**FIGURE 9:**
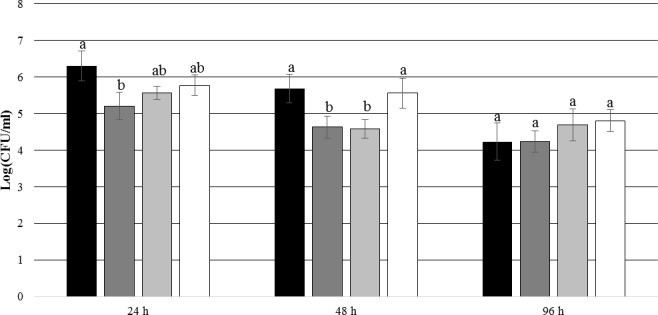
Number of attached *P. inhibens* wt with either *E. huxleyi* or f/2 medium inside the chamber. Grouping based on ANOVA *post hoc* Tukey test. Groups within each sampling time that do not share a letter are significantly different (24 h: *P* = 0.018; 48 h: *P* = 0.007; 96 h: *P* = 0.220). 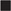 *P. inhibens* wt with *E. huxleyi*, experimental membranes; 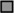 *P. inhibens* wt with *E. huxleyi*, blinded membranes; 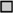 *P. inhibens* wt with f/2 medium, experimental membranes; 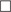 *P. inhibens* wt with f/2 medium, control membranes.

**FIGURE 10:**
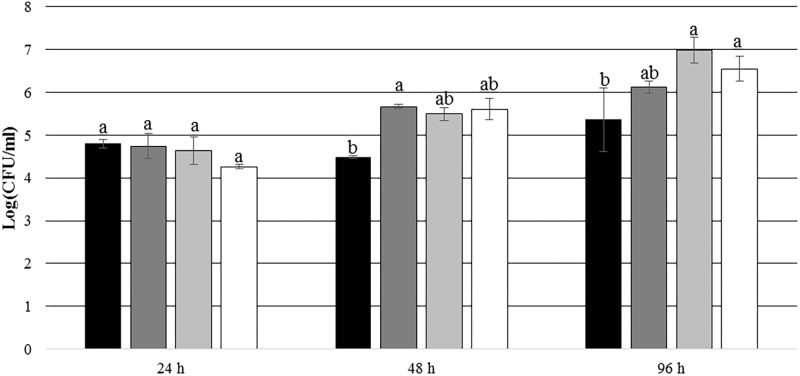
Number of attached *P. inhibens* Δ*tdaB* from 24 to 96 h with either *E. huxleyi* or f/2 medium inside the chamber. Grouping based on ANOVA *post hoc* Tukey test. Groups within each sampling time that do not share a letter are significantly different (24 h: *P* = 0.068; 48 h: *P* = 0.024; 96 h: *P* = 0.010). 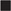 *P. inhibens* *ΔtdaB* with *E. huxleyi*, experimental membranes; 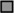 *P. inhibens* *ΔtdaB* with *E. huxleyi*, blinded membranes; 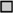 *P. inhibens* *ΔtdaB* with f/2 medium, experimental membranes; 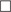 *P. inhibens* *ΔtdaB* with f/2 medium, control membranes.

After 24 h of incubation, *P. inhibens* wt was found to be attached to the experimental membranes in higher numbers than to the control membranes when *E. huxleyi* was present inside the culture chamber (*p* = 0.004) (**Figure 9**). After 48 and 96 h of incubation, no such difference could be seen (*p* = 0.097 for 48 h and *p* = 0.971 for 96 h). This indicates that the presence of *E. huxleyi* positively affects initial attachment of *P. inhibens* wt. There were no significant trends in attachment of *P. inhibens* Δ*tdaB* over time, except than less cells were attached to the experimental membranes after 48 h with *E. huxleyi* inside the culture chamber (*p* = 0.001) (**Figure 10**).

When the chamber content was sterile f/2 medium, there were no significant differences in attachment to the experimental and the control membranes of neither the wt or the TDA-negative mutant strain (24 h/wt: *p* = 0.322; 24 h/Δ*tdaB*: *p* = 0.179; 48 h/wt: *p* = 0.052; 48 h/Δ*tdaB*: *p* = 0.498; 96 h/wt: *p* = 0.797; 96 h/Δ*tdaB*: *p* = 0.128), which further confirmed that the presence of *E. huxleyi* did affect attachment and biofilm formation of *P. inhibens*.

### Method Validation and Improvements

One source of error during set up of the culture chambers was leakage due to improper assembly or cracked PAO membranes. Two approaches were taken to address with this. The first was the use of a 0.6 mm thick Teflon ring between the culture chamber and outer membrane, acting as a washer to provide a better seal during assembly. The second was the use of microbeads as a control for leakage. When chambers were loaded with 0.5 μM diameter fluorescein labeled polycarbonate beads, the recovery (judged by microscopy of 5 μl volumes) was >60% after incubation for a week. In deliberately sabotaged chambers by puncturing the PAO membrane, recovery was <10%. Whilst the user will need to calibrate these cut-off value for themselves, depending on the membrane porosity and adherence properties, we recommend this approach to remove invalid outliers from data sets. Using cefataxime, rifampicin and fluorescein as tracers we have shown that these molecules are able to diffuse into and out of the culture chamber freely. We note that TDA is a smaller molecule and has no tendency to bind to any of the surfaces used within the experimental set up, including polycarbonate membranes. This suggests that TDA was also able to exchange across the experimental membrane during co-culture. Finally, it is noted that experiments in which the inner chamber is loaded with nutrients depends on an appropriate rate of diffusion through the chosen membrane; too slow and too little reaches the microbiota in the bulk phase; too fast and the nutrient gradient, and therefore the impact may be transient.

## Discussion

The microbial culture chamber proved well suited for studying the effect of co-culturing of microorganisms, and particularly their biofilm formation, in a highly defined *in vitro* co-culture setup. Other chambers, the iChip (Nichols et al., 2010) and the diffusion growth chamber (Kaeberlein et al., 2002) are essentially instruments for environmental co-culturing where microorganisms are cultured inside the same chamber, which is suitable for studying enrichment cultures and for increasing culturability of rare and slow-growing or otherwise intractable microorganisms. However, these chambers are not suited for experimental work *in situ*, i.e., detailed investigation of the effects of one specific organism or co-factor on another. Instead, the microbial culture chamber presented here is unusual in that it offers an *in vitro* as well as an *in situ* experimental setup, where it is possible to measure the effect of enrichment or co-culturing by, e.g., quantifying the number of attached bacteria or by investigating the diversity of attached bacteria in a mixed community as a response to the content of the inner chamber.

Here we developed a method and setup to determine how the contents of a deployable culture chamber (a microorganism or a macroalgae acting as a nutrient source) affects bacteria on the outer surface and potentially influence biofilm development. We note that the method is versatile, allowing use of two types of membrane and bacterial quantification by DNA analysis or microscopy. The latter point suggests utility in future metagenomics, transcriptomics or proteomics studies. The method requires exchange of soluble compounds through one or more membranes, with the number of membranes fine tuning the rate of diffusion.

The chamber was used here to study a well characterized interaction between algae and bacteria. Attachment and biofilm formation has been observed in many species of marine bacteria (Mai-Prochnow, 2004; Rao et al., 2005; Doghri et al., 2015; Beleneva et al., 2017). It has recently been shown that *P. inhibens* physically interact with *E. huxleyi* during algal blooms (Segev et al., 2016), however, it was not shown whether the attachment was directly induced by the presence of the algae, even though Segev et al. (2016) did show that attachment of *P. inhibens* did indeed promote growth of the algae through production of the potential growth hormone indole-3-acetic acid making such a close association favorable to both bacteria and algae.

Our microscopy data indicated that there was a correlation between the close proximity and access to *E. huxleyi* and the attachment and biofilm formation of *P. inhibens*. Quantitative PCR was used to document the quantitative difference in the number of bacteria attached to membranes used to separate the bacterial culture from the algal culture. There was indeed was a significant difference between the number of cells attached to membranes with access to *E. huxleyi* compared to membranes blinded from the algal culture by a metal plate or to membranes exposed to f/2 medium. We also found that the numbers of attaching bacteria differed between the wt strain and the TDA knock out mutant.

*Emiliania huxleyi* is a phototrophic alga and potentially growth would stop, and senescence would start shortly after being enclosed in the culture chamber, since day light has a proven effect on the growth of *E. huxleyi* (Nielsen, 1997). Given, the observed differences of bacterial attachment between the experimental and control membranes, the alga clearly secretes sufficient metabolites to be detectable by the bacteria. We note that the central chamber is not completely dark (low levels of light can penetrate through the experimental membrane) which may have contributed to the success of these experiments. However, as engineering grade plastics exist that are transparent we would propose changing future chambers to allow illumination of the interior.

In this study, we included a TDA-deletion strain of *P. inhibens* DSM17395 to test whether TDA production would affect its attraction to and attachment in the presence of *E. huxleyi*. The production of TDA limits growth and biomass production in TDA-deficient *P. inhibens* DSM17395 as compared to the wt strain (Will et al., 2017). TDA-deficient strains reached a maximum biomass yield after 20 h when the carbon source was depleted, whereas TDA-producing strains entered stationary phase after 30 h despite carbon source still remaining the medium and a lower total biomass yield (Will et al., 2017). This could possibly explain why we detected a higher number of *P. inhibens* Δ*tdaB* cells attached to the culture chamber membranes after 96 h if growth of the wt strain was inhibited between 24 and 48 h after inoculation, whereas carbon source might have been sufficient to keep the TDA-mutant in an active growth phase beyond 48 h of incubation.

During the first 24 h of incubation, we expected *P. inhibens* Δ*tdaB* to grow faster to higher densities than the wt strain. However, the wt strain attached in higher numbers to the culture chamber membranes when *E. huxleyi* was present inside the chamber indicating an active interaction between the algae, the TDA production capability and the biofilm forming ability of the bacterium. This is in line with previous observations that biofilm formation coincides with TDA production (Bruhn et al., 2007; Geng et al., 2008). More *P. inhibens* wt cells attached to the experimental membranes with access to *E. huxleyi* compared to attachment of the TDA-mutant strain within the first 48 h despite expected slower growth rate of the wt. Since the wt strain attached in higher numbers to membranes with access to *E. huxleyi* than to control membranes where there was no access to the alga, we believe that the higher level of attachment of the TDA-producing wt strain is more likely to be due to the presence of *E. huxleyi* than to the ability to produce TDA itself.

In summary, we have developed a novel, versatile co-cultivation method to study interaction of microorganisms *in situ* as well as *in vitro* using a microbial culture chamber. We show that the culture chamber can be used for enrichment through microbial interactions, and that the presence of a specific microorganism, *E. huxleyi*, affects attachment of *P. inhibens* to polycarbonate membranes.

## Author Contributions

MT and LG came up with the experiments for algal-bacterial co-culturing. MT and JM conducted the experiments on the *Phaeobacter-Emiliania* co-culturing. CI invented and produced the biochamber and conducted the Fucus-enrichment experiments. MT performed statistical analysis. MT, CI, and LG drafted the manuscript.

## Conflict of Interest Statement

CI is employed by Hoekmine BV, which has a commercial interest in the microbial culture chambers and cultivation methods. The remaining authors declare that the research was conducted in the absence of any commercial or financial relationships that could be construed as a potential conflict of interest.

## References

[B1] Beleneva I. A. Skriptsova A. V. Svetashev V. I. (2017). Characterization of biofilm-forming marine bacteria and their effect on attachment and germination of algal spores. *Microbiology* 86 317–329. 10.1134/S0026261717030031

[B2] Bernbom N. Ng Y. Y. Kjelleberg S. Harder T. Gram L. (2011). Marine bacteria from Danish coastal waters show antifuling activity against the marine fouling bacterium *Pseudoalteromonas* sp. strain S91 and zoospores of the green alga *Ulva Australis* independent of bacteriocidal activity. *Appl. Environ. Microbiol.* 77 8557–8567. 10.1128/AEM.06038-11 22003011 PMC3233102

[B3] Boon E. Meehan C. J. Whidden C. Wong D. H. Langille M. G. Beiko R. G. (2014). Interactions in the microbiome: communities of organisms and communities of genes. *FEMS Microbiol. Rev.* 38 90–118. 10.1111/1574-6976.12035 23909933 PMC4298764

[B4] Boström K. H. Simu K. Hagström Å Riemann L. (2004). Optimization of DNA extraction for quantitative marine bacterioplankton community analysis. *Limnol. Oceanogr. Methods* 2 365–373. 10.4319/lom.2004.2.365

[B5] Bruhn J. B. Gram L. Belas R. (2007). Production of antibacterial compounds and biofilm formation by *Roseobacter* species are influenced by culture conditions. *Appl. Environ. Microbiol.* 73 442–450. 10.1128/AEM.02238-06 17098910 PMC1796973

[B6] Catón L. Yurkov A. Giesbers M. Dijksterhuis J. Ingham C. J. (2017). Physically triggered morphology changes in a novel *Acremonium isolate* cultivated in precisely engineered microfabricated environments. *Front. Microbiol.* 8:1269. 10.3389/fmicb.2017.01269 28769882 PMC5509762

[B7] D’Alvise P. W. Lillebø S. Prol-Garcia M. J. Wergeland H. I. Nielsen K. F. Bergh Ø (2012). *Phaeobacter gallaeciensis* reduces *Vibrio anguillarum* in cultures of microalgae and rotifers, and prevents vibriosis in cod larvae. *PLoS One* 7:e43996. 10.1371/journal.pone.0043996 22928051 PMC3425499

[B8] Doghri I. Rodrigues S. Bazire A. Dufour A. Akbar D. Sopena V. (2015). Marine bacteria from the French Atlantic coast displaying high forming-biofilm abilities and different biofilm 3D architectures. *BMC Microbiol.* 15:231. 10.1186/s12866-015-0568-r4 26498445 PMC4619314

[B9] Finkelshtein A. Roth D. Ben Jacob E. Ingham C. J. (2015). Bacterial swarms recruit cargo bacteria to pave the way in toxic environments. *mBio* 6:e00074-15. 10.1128/mBio.00074-r15 25968641 PMC4436059

[B10] Geng H. Bruhn J. B. Nielsen K. F. Gram L. Belas R. (2008). Genetic dissection of tropodithietic acid biosynthesis by marine roseobacters. *Appl. Environ. Microbiol.* 74 1535–1545. 10.1128/AEM.02339-07 18192410 PMC2258615

[B11] Goers L. Freemont P. Polizzi K. M. (2014). Co-culture systems and technologies: taking synthetic biology to the next level. *J. R. Soc. Interface* 11:20140065. 10.1098/rsif.2014.0065 24829281 PMC4032528

[B12] Gram L. Rasmussen B. B. Wemheuer B. Bernbom N. Ng Y. Y. Porsby C. H. (2015). *Phaeobacter inhibens* from the *Roseobacter clade* has an environmental niche as a surface colonizer in harbors. *Syst. Appl. Microbiol.* 38 483–493. 10.1016/j.syapm.2015.07.006 26343311

[B13] Grotkjær T. Bentzon-Tilia M. D’Alvise P. Dierckens K. Bossier P. Gram L. (2016). *Phaeobacter inhibens* as probiotic bacteria in non-axenic Artemia and algae cultures. *Aquaculture* 462 64–69. 10.1016/j.aquaculture.2016.05.001

[B14] Guillard R. R. L. (1975). *“Culture of phytoplankton for feeding marine invertebrates,” in Culture of Marine Invertebrate Animals*, eds Smith W. L. Chanley M. H. (Boston, MA: Springer), 29–60. 10.1007/978-1-4615-8714-9_3

[B15] Guillard R. R. L. Ryther J. H. (1962). Studies of marine planktonic diatoms. I. cyclotella nana hustedt and detunola confervacea (Cleve) Gran. *Can. J. Microbiol.* 8 229–239. 10.1139/m62-029 13902807

[B16] Hay W. W. Mohler H. P. Roth P. H. Schmidt R. R. Boudreaux J. E. (1967). Calcareous nannoplankton zonation of the Cenozoic of the Gulf Coast and Caribbean-Antillean area, and transoceanic correlation. *Trans. Gulf Coast Assoc. Geol. Soc.* 17 428–480.

[B17] Ingham C. J. Sprenkels A. Bomer J. Molenaar D. van den Berg A. Vlieg J. E. T. (2007). The micro-Petri dish, a million-well growth chip for the culture and high-throughput screening of microorganisms. *Proc. Natl. Acad. Sci. U.S.A.* 104 18217–18222. 10.1073/pnas.0701693104 17989237 PMC2084323

[B18] Ingham C. J. van Hylckama Vlieg J. E. (2008). MEMS and the microbe. *Lab Chip* 8 1604–1616. 10.1039/B8047960A 18813380

[B19] Kaeberlein T. Lewis K. Epstein S. S. (2002). Isolating “uncultivable” microorganisms in pure pulture in a simulated natural environment. *Science* 296 1127–1129. 10.1126/science.1070633 12004133

[B20] Lachnit T. Fischer M. Künzel S. Baines J. F. Harder T. (2013). Compounds associated with algal surfaces mediate epiphytic colonization of the marine macroalga *Fucus vesiculosus*. *FEMS Microbiol. Ecol.* 84 411–420. 10.1111/1574-6941.12071 23311942

[B21] Lachnit T. Wahl M. Harder T. (2010). Isolated thallus-associated compounds from the macroalga *Fucus vesiculosus* mediate bacterial surface colonization in the field similar to that on the natural alga. *Biofouling* 26 247–255. 10.1080/08927010903474189 20054721

[B22] L’Haridon S. Markx G. H. Ingham C. J. Paterson L. Duthoit F. Le Blay G. (2016). “New approaches for bringing the Uncultured into Culture,” in *The Marine Microbiome: An Untapped Source of Biodiversity and Biotechnological Potential*, eds Stal L. J. Cretoiu M. S. (Cham: Springer), 401–434.

[B23] Ling L. L. Schneider T. Peoples A. J. Spoering A. L. Engels I. Conlon B. P. (2015). A new antibiotic kills pathogens without detectable resistance. *Nature* 517 455–459. 10.1038/nature14098 25561178 PMC7414797

[B24] Lutz C. Thomas T. Steinberg P. Kjelleberg S. Egan S. (2016). Effect of interspecific competition on trait variation in *Phaeobacter inhibens* biofilms. *Environ. Microbiol.* 18 1635–1645. 10.1111/1462-2920.13253 26914307

[B25] Mai-Prochnow A. (2004). Biofilm development and cell death in the marine bacterium *Pseudoalteromonas tunicata.* *Appl. Environ. Microbiol.* 70 3232–3238. 10.1128/AEM.70.6.3232-3238.2004 15184116 PMC427794

[B26] Martens T. Heidorn T. Pukall R. Simon M. Tindall B. J. Brinkhoff T. (2006). Reclassification of *Roseobacter gallaeciensis* Ruiz-Ponte et al., 1998 as *Phaeobacter gallaeciensis* gen. nov., comb. nov., description of *Phaeobacter inhibens* sp. nov., reclassification of *Ruegeria algicola* (Lafay et al., 1995) Uchino et al., 1999 as *Marinovu algicola* gen. nov., comb. nov., and emended description of the genera *Roseobacter, Ruegeria* and *Leisingera*. *Int. J. Syst. Evol. Microbiol.* 56 1293–1304. 10.1099/ijs.0.63724-0 16738106

[B27] Morris J. J. Kirkegaard R. Szul M. J. Johnson Z. I. Zinser E. R. (2008). Facilitation of robust growth of *Prochlorococcus colonies* and dilute liquid cultures by “helper” heterotrophic bacteria. *Appl. Environ. Microbiol.* 74 4530–4534. 10.1128/AEM.02479-07 18502916 PMC2493173

[B28] Nichols D. Cahoon N. Trakhtenberg E. M. Pham L. Mehta A. Belanger A. (2010). Use of ichip for high-throughput in situ cultivation of “uncultivable” microbial species. *Appl. Environ. Microbiol.* 76 2445–2450. 10.1128/AEM.01754-09 20173072 PMC2849220

[B29] Nielsen M. V. (1997). Growth, dark respiration and photosynthetic parameters of the coccolithophorid Emiliania huxleyi (*Prymnesiophyceae*) acclimated to different day-length-irradiance combiations. *J. Phycol.* 33 818–822. 10.1111/j.0022-3646.1997.00818.x

[B30] Park J. Kerner A. Burns M. A. Lin X. N. (2011). Microdroplet-enabled highly parallel co-cultivation of microbial communities. *PLoS One* 6:e17019. 10.1371/journal.pone.0017019 21364881 PMC3045426

[B31] Porsby C. H. Gram L. (2016). *Phaeobacter inhibens* as biocontrol agent against *Vibrio vulnificus* in oyster models. *Food Microbiol.* 57 63–70. 10.1016/j.fm.2016.01.005 27052703

[B32] Prol M. J. Bruhn J. B. Pintado J. Gram L. (2009). Real-time PCR detection and quantification of fish probiotic *Phaeobacter* strain 27-4 and fish pathogenic *Vibrio* in microalgae, rotifer, Artemia and first feeding turbot (*Psetta maxima*) larvae. *J. Appl. Microbiol.* 106 1292–1303. 10.1111/j.1365-2672.2008.04096.x 19187159

[B33] Prol García M. J. D’Alvise P. W. Rygaard A. M. Gram L. (2014). Biofilm formation is not a prerequisite for production of the antibacterial compound tropodithietic acid in *Phaeobacter inhibens* DSM17395. *J. Appl. Microbiol.* 117 1592–1600. 10.1111/jam.12659 25284322

[B34] Ramanan R. Kim B.-H. Cho D.-H. Oh H.-M. Kim H.-S. (2016). Algae-bacteria interactions: evolution, ecology and emerging applications. *Biotechnol. Adv.* 34 14–29. 10.1016/j.biotechadv.2015.12.003 26657897

[B35] Rao D. Webb J. S. Kjelleberg S. (2005). Competitive interactions in mixed-species biofilms containing the marine bacterium *Pseudoalteromonas tunicata*. *Appl. Environ. Microbiol.* 71 1729–1736. 10.1128/AEM.71.4.1729-1736.2005 15811995 PMC1082554

[B36] Saha M. Rempt M. Grosser K. Pohnert G. Weinberger F. (2011). Surface-associated fucoxanthin mediates settlement of bacterial epiphytes on the rockweed *Fucus vesiculosus*. *Biofouling* 27 423–433. 10.1080/08927014.2011.580841 21547758

[B37] Schneider C. A. Rasband W. S. Eliceiri K. W. (2012). NIH image to imageJ: 25 years of image analysis. *Nat. Methods* 9 671–675. 10.1038/nmeth.2089 22930834 PMC5554542

[B38] Segev E. Castañeda I. S. Sikes E. L. Vlamakis H. Kolter R. (2015). Bacterial influence on alkenones in live microalgae. *J. Phycol.* 52 125–130. 10.1111/jpy.12370 26987094 PMC4800829

[B39] Segev E. Wyche T. P. Kim K. H. Petersen J. Ellebrandt C. Vlamakis H. (2016). Dynamic metabolic exchange governs a marine algal-bacterial interaction. *eLife* 5:e17473. 10.7554/eLife.17473 27855786 PMC5148602

[B40] Seyedsayamdost M. R. Carr G. Kolter R. Clardy J. (2011a). *Roseobacticides*: small molecule modulators of an algal-bacterial symbiosis. *J. Am. Chem. Soc.* 133 18343–18349. 10.1021/ja207172s 21928816 PMC3211371

[B41] Seyedsayamdost M. R. Case R. J. Kolter R. Clardy J. (2011b). The Jekyll-and-Hyde chemistry of *Phaeobacter gallaeciensis*. *Nat. Chem.* 3 331–335. 21430694 10.1038/nchem.1002 PMC3376411

[B42] Seyedsayamdost M. R. Wang R. Kolter R. Clardy J. (2014). Hybrid biosynthesis of roseobacticides from algal and bacterial precursor molecules. *J. Am. Chem. Soc.* 136 15150–15153. 10.1021/ja508782y 25295497 PMC4227733

[B43] Shi Y. Pan C. Wang K. Chen X. Wu X. Chen C.-T. A. (2017). Synthetic multispecies microbial communities reveals shifts in secondary metabolism and facilitates cryptic natural product discovery. *Environ. Microbiol.* 19 3606–3618. 10.1111/1462-2920.13858 28714207

[B44] Sobecky P. A. Mincer T. J. Chang M. C. Helinski D. R. (1997). Plasmids isolated from marine sediment microbial communities contain replication and incompatibility regions unrelated to those of known plasmid groups. *Appl. Environ. Microbiol.* 63 888–895. 9055407 10.1128/aem.63.3.888-895.1997 PMC168381

[B45] Sonnenschein E. C. Phippen C. Bentzon-Tilia M. Rasmussen S. A. Nielsen K. F. Gram L. (2018). Roseobacter gardening of microalgae: phylogenetic distribution of roseobacticides and their effect on microalgae. *Environ. Microbiol. Rep.* 10 383–393. 10.1111/1758-2229.12649 29624899

[B46] Stewart E. J. (2012). Growing unculturable bacteria. *J. Bacteriol.* 194 4151–4160. 10.1128/JB.00345-12 22661685 PMC3416243

[B47] Wahl M. (1989). Marine epibiosis. I. Fouling and antifoulinng: some basic aspects. *Mar. Ecol. Prog. Ser.* 58 175–189. 10.3354/meps058175

[B48] Wakefield J. Hassan H. M. Jaspars M. Ebel R. Rateb M. E. (2017). Dual induction of new microbial secondary metabolites by fungal bacterial co-cultivation. *Front. Microbiol.* 8:1284. 10.3389/fmicb.2017.01284 28744271 PMC5504103

[B49] Wang R. Gallant É Seyedsayamdost R. (2016). Investigation of the genetics and biochemistry of roseobacticide production in the *Roseobacter clade* bacterium *Phaeobacter inhibens*. *mBio* 7:e02118. 10.1128/mBio.02118-15 27006458 PMC4807370

[B50] Wang R. Seyedsayamdost M. R. (2017). Roseochelin B, an algaecidal natural product synthesized by the roseobacter *Phaeobacter inhibens* in response to algal sinapic acid. *Org. Lett.* 19 5138–5141. 10.1021/acs.orglett.7b02424 28920692

[B51] Will S. E. Neumann-Schaal M. Heydorn R. L. Bartling P. Petersen J. Schomburg D. (2017). The limits to growth – energetic burden of the endogenous antibiotic tropodithietic acid in *Phaeobacter inhibens* DSM 17395. *PLoS one* 12:e0177295. 10.1371/journal.pone.0177295 28481933 PMC5421792

[B52] Zhao W. Dao C. Karim M. Gomez-Chiarri M. Rowley D. Nelson D. R. (2016). Contributions of tropodithietic acid and biofilm formation to the probiotic activity of *Phaeobacter inhibens*. *BMC Microbiol.* 16:1. 10.1186/s12866-015-0617-z 26728027 PMC4700733

